# Chinese Herbal Medicine (MaZiRenWan) Improves Bowel Movement in Functional Constipation Through Down-Regulating Oleamide

**DOI:** 10.3389/fphar.2019.01570

**Published:** 2020-01-23

**Authors:** Tao Huang, Ling Zhao, Cheng-Yuan Lin, Lin Lu, Zi-Wan Ning, Dong-Dong Hu, Linda L. D. Zhong, Zhi-Jun Yang, Zhao-Xiang Bian

**Affiliations:** ^1^ Institute of Brain and Gut Research, School of Chinese Medicine, Hong Kong Baptist University, Hong Kong, Hong Kong; ^2^ YMU-HKBU Joint Laboratory of Traditional Natural Medicine, Yunnan Minzu University, Kunming, China; ^3^ Hong Kong Chinese Medicine Clinical Study Centre, Hong Kong Baptist University, Hong Kong, Hong Kong

**Keywords:** MaZiRenWan, functional constipation, oleamide, pharmacometabolomics, fatty acid amide hydrolase

## Abstract

In a prospective, randomized, three-arms, controlled clinical study, Chinese Herbal Medicine MaZiRenWan (MZRW, also known as Hemp Seed Pill) demonstrates comparable efficacy with Senna for functional constipation (FC) during an 8-week treatment period. Both MZRW and Senna are better than a placebo; relative to Senna and a placebo, MZRW displayed a more sustained effect during the 8-week follow-up period. The characteristic pharmacological mechanism responsible for this observation is still unclear. To explore this, we collected pre- and post-treatment serum samples of 85 FC patients from MZRW/Senna/placebo treatment groups for pharmacometabolomic analysis. An ultrahigh-performance liquid chromatography-mass spectrometer (UPLC-MS) was used for metabolic profiling and quantification. *In vivo* studies were conducted in constipated C57BL/6J mice to verify the effects and corresponding mechanism(s) of the action of MZRW. Pearson correlation analysis, paired t-test, one-way ANOVA analysis, χ2 test, and Student t-test were used to interpret the clinical and preclinical data. Changes in levels of circulating oleamide and its derivatives negatively correlate with improvement in complete spontaneous bowel movement (CSBM) in the MZRW group (Pearson r = -0.59, *p* = 0.00057). The same did not hold true for either Senna or placebo groups. Oleamide is a known regulator of intestinal motility. MZRW treatment resulted in reduced levels of circulating oleamide in FC patients. Experimental verification showed that MZRW attenuated oleamide-induced slow intestinal motility in mice. MZRW decreased oleamide levels in serum, ileum, and colon in normal mice, but increased expression of colonic fatty acid amide hydrolase (FAAH). In conclusion, MZRW improved bowel movement in FC by down-regulating oleamide, possibly by enhancing FAAH-mediated degradation. Our findings suggest a novel therapeutic strategy for FC.

## Introduction

Functional constipation (FC) affects over 14% of adults globally (Suares and Ford, 2011). Women, the elderly, or people with lower socioeconomic status are more likely to be affected by FC (Suares and Ford, 2011). FC significantly impacts patients’ quality of life and causes heavy societal burden (Liem et al., 2009; Peery et al., 2012; Peery et al., 2015). Patients with mild or moderate FC can easily be treated with high-fiber foods or laxatives (Rao et al., 2015; Christodoulides et al., 2016), while those with severe FC require special care and impactful treatments, including anthraquinone, diphenyl methanes or derivatives, 5-hydroxytryptamine receptor 4 agonist, guanylate cyclase C receptor agonist, chloride channel type 2 activator, apical sodium bile acid inhibitors, etc. (Nelson et al., 2016). More than 50% of FC patients are not completely satisfied with their current therapies (Nelson et al., 2016), thus alternative treatment approaches are needed.

MaZiRenWan (MZRW, also known as Hemp Seed Pill), an herbal formula from Traditional Chinese Medicine (TCM), has been used for constipation for about two millennia (Zhong et al., 2016). It is comprised of six herbal ingredients, including *Cannabis Fructus* (plant source: *Cannabis sativa L*.), *Rhei Radix et Rhizoma* (plant source: *Rheum tanguticum* Maxim. ex Regel) Balf., *Armeniacae Semen Amarum* (plant source: *Prunus mandshurica* (Maxim.) Koehne), *Paeoniae Radix Alba* (plant source: *Paeonia lactiflora* Pall.), *Magnoliae Officinalis Cortex* (plant source: *Magnolia officinalis* Rehder & E.H.Wilson), and *Aurantii Fructus Immaturus* (plant source*: Citrus aurantium* L.) (Cheng et al., 2011). A systematic review revealed that it is the most commonly used herbal medicine for constipation in China (Zhong et al., 2016). Our group has conducted several clinical studies to validate the efficacy and safety of MZRW in FC patients. MZRW has been demonstrated to be more effective than a placebo in a phase I/II study with 120 FC patients (Cheng et al., 2011). Using a network pharmacology approach, 5 active compounds were identified in MZRW:emodin, amygdalin, albiflorin, honokiol, and naringin. These compounds have been shown to stimulate the contractions of colonic smooth muscle through multiple signaling pathways, such as the acetylcholine-, estrogen-, prostaglandin-, cannabinoid-, and purine pathways (Huang et al., 2018). Recently, in a three-armed, randomized, and controlled clinical study with 291 FC patients (Zhong et al., 2019), MZRW demonstrated comparable efficacy with Senna (a first-line laxative commonly used in Hong Kong) during the treatment period. Both were better than a placebo. MZRW had a more sustained effect during the follow-up period than either Senna or a placebo. The results suggest that MZRW may alleviate FC through distinct mechanisms-of-action.

Encouraging clinical results led to the design and conduction of a pharmacometabolomic analysis and pharmacological study. Pharmacometabolomics involve mapping drug effects on metabolism and identifying pathways which contribute to drug efficacy and/or adverse drug reactions (Kaddurah-Daouk and Weinshilboum, 2014). By analyzing the pre- and post-treatment serum samples from 85 FC patients, who were randomly selected from the three-armed groups in the three-armed clinical study, we found a representative metabolite identified as oleamide to be highly related to intestinal motility. The change of serum oleamide is significantly correlated with complete spontaneous bowel movement (CSBM) improvement in the MZRW group, but not with CSBM improvement in the Senna or placebo group. This suggests that oleamide signaling could be specifically targeted by MZRW to relieve FC. How oleamide is regulated by MZRW in FC patients and mice was then investigated.

## Materials and Methods

### Participants

Participants were eligible as per the published protocol (Zhong et al., 2013) and clinical study (Zhong et al., 2019). In brief, subjects were included if the following requirements were met: 1) diagnosed with FC according to Rome III; 2) diagnosed with excessive pattern according to TCM; 3) 18-65 years old; 4) complete spontaneous bowel movement (CSBM) ≤ 2times/week; 5) severity of constipation ≥ 3 points (on a 7-point scale) (Ford and Suares, 2011); 6) total symptom score ≥ 8 points (on a 7-point scale for constipation-related symptoms); 7) normal colonic examination (barium enema or colonoscopy) within five years; 8) normal liver and renal function in blood test within 3 months. Subjects were excluded if they had 1) secondary constipation; 2) severe diseases; 3) undergone abdominal surgery within one year; 4) a history of allergy to Chinese herbal medicine; 5) psychiatric or addictive disorders requiring medications with side effects of constipation. Pregnant or lactating women were also excluded. CSBM was chosen as the major endpoint of efficacy. The final number of patients included from different intervention group in this study was: MZRW, 30; Senna, 31; placebo, 24.

### Preparation of MZRW Granules for FC Patient’s Intervention

Details on MZRW preparation for FC patients have been documented in a previously published clinical study (Zhong et al., 2019). In brief, a dose of 15 g per day (7.5 g, twice a day) of MZRW granules was selected. Both MZRW and placebo granules were prepared by PuraPharm Pharmaceuticals (Nanning, China), and the standards of Good Manufactory Practice and Chinese Pharmacopoeia were strictly complied with during the manufacturing process. A sachet of granules was dissolved in 150 mL of hot water, and taken orally twice a day by patients. The placebo granules comprised of dextrin (76.03%), tea essence (23.61%), gardenin (0.02%), and caramel (0.34%) (Cheng et al., 2011). Voucher numbers of individual specimens of MZRW were given (**Table S1**) and samples were kept at the School of Chinese Medicine, Hong Kong Baptist University, Hong Kong. *Cannabis Fructus* (35.7%), *Rhei Radix et Rhizoma* (17.9%), *Armeniacae Semen Amarum* (17.9%), *Paeoniae Radix Alba* (8.9%), *Magnoliae Officinalis Cortex* (10.7%), and *Aurantii Fructus Immaturus* (8.9%) were included in the MZRW granules. The full chemical composition of MZRW granules was identified by UPLC-Q-TOF-MS (**Table S2**), and major compounds were quantified: amygdalin (11.03 ± 0.15 mg/g), albiflorin (0.56 ± 0.01 mg/g), paeoniflorin (1.3 ± 0.03 mg/g), naringin (0.67 ± 0.01 mg/g), hesperidin (12.25 ± 0.13 mg/g), aloe emodin (1.53 ± 0.02 mg/g), rhein (18.23 ± 0.10 mg/g), emodin (6.15 ± 0.03 mg/g), honokiol (6.87 ± 0.05 mg/g), and magnolol (1.24 ± 0.02 mg/g). The Senna tablets (Senokot) containing 7.5 mg sennoside B, were purchased from Reckitt Benckiser (Slough, UK). The placebo tablets comprised of starch and pigment (Guangzhou Huahai Pharmaceuticals, Guangzhou, China).

### Preparation of MZRW Extract for Animal Study

The preparation of the MZRW extract was similar to the previous study (Huang et al., 2018). *Cannabis Fructus* (892.9 g), *Rhei Radix et Rhizoma* (446.4g), *Armeniacae Semen Amarum* (446.4 g), *Paeoniae Radix Alba* (223.2 g), *Magnoliae Officinalis Cortex* (267.9 g), and *Aurantii Fructus Immaturus* (223.2 g) were mixed and decocted with 8-flood volumes of water (1:8, w/v) for 2.5 h. The decoction was then filtered. Concentrated decoction was prepared by rotary evaporation under vacuum at 55 °C. The residue was freeze dried. Finally, the dried residue was dissolved in water to get an oral solution of concentration 0.5 g/mL. Raw materials were stored at room temperature, while MZRW preparations were stored at -20 °C. The full chemical composition of the MZRW extract was identified by UPLC-Q-TOF-MS (**Table S3**). Major compounds of the MZRW extract were quantified by LC-MS: amygdalin (9.91 ± 0.55 mg/g), albiflorin (0.70 ± 0.07 mg/g), paeoniflorin (1.37 ± 0.07 mg/g), naringin (0.70 ± 0.06 mg/g), hesperidin (12.23 ± 0.46 mg/g), aloe emodin (1.61 ± 0.11 mg/g), rhein (17.72 ± 1.46 mg/g), emodin (6.51 ± 0.30 mg/g), honokiol (7.23 ± 0.09 mg/g), and magnolol (1.30 ± 0.10 mg/g).

### Untargeted Serum Metabolomics Profiling

Sample preparation was continued, referring to standard methods for metabolic profile (Álvarez-Sánchez et al., 2010a; Álvarez-Sánchez et al., 2010b). Organic reagents (MS grade) for liquid chromatography tandem mass spectrum (LC-MS) analysis were purchased from Burdick & Jackson (Muskegon, MI, USA)., A quadruple volume of methanol was added into samples, vortex-mixed for 30s and centrifuged at 12000 rpm for 10 min at 4°C. The supernatant was then transferred to new tubes with 10 μL of internal standard (IS, 0.1 mg mL^−1^ of 4-chlorophenylalanine solution) for LC-MS analysis. Quality control samples were prepared from a pooled mixture equally derived from all samples and preprocessed following the same protocol. An ultrahigh-performance liquid chromatography (UPLC, Agilent 1290 Infinity, USA) coupled to electrospray ionization (ESI) quadrupole time-of-flight mass spectrometer (Q-TOF MS, Agilent 6543, USA) was employed for metabolic profiling. A Waters ACQUITY UPLC BEH C18 column (1.7μm, 2.1mm×50mm, Waters Corporation, Milford, MA) was operated for serum metabolite separation. A 2-μL aliquot of each sample was injected into the column, while temperature was maintained at 40°C. The mobile phase consisted of water containing 0.1% formic acid (A) and acetonitrile containing 0.1% formic acid (B). The gradient program was from 30% B to 100% B with 0.35 mL min^−1^ of flow rate within 10 min. Meanwhile, mass spectrometry was performed in both positive and negative ion modes with a gas temperature of 300°C, drying gas flow rate of 8 L min^−1^, capillary voltage at 3000 V, and fragment voltage at 150 V. The *m/z* range of acquisition was set at 80 to 1000.

### Quantitative Analysis of Oleamide in Specimens

#### Oleamide Extraction

The procedure of oleamide extraction in specimens was similar to that of a previous study (Schreiber et al., 2007). Briefly, the 200 μL volume of acetone was mixed with 200 μL of serum, and precisely weighed tissues (50 mg) were homogenized with a five-fold volume of acetone for protein precipitation. After low temperature centrifugation (12000 rpm for 10 min), supernatants were collected and dried under a stream of nitrogen. Subsequently, oleamide was extracted with a mixture of chloroform and methanol (2:1, v/v), and chloroform phases were evaporated to dryness under nitrogen, reconstituted in acetonitrile, and finally transferred to sampling tubes for further LC-MS analysis.

#### LC-MS Instrumental Conditions

The detection was conducted using a liquid chromatography (Agilent LC 1290, USA) coupled to a triple-quadrupole mass spectrometer (Agilent QQQ-MS 6438, USA) in positive ESI mode with multiple reaction monitoring (MRM). 5 µL of each sample was injected onto a UPLC BEH C18 column and eluted using a linear gradient of deionized water (A) and acetonitrile (B) with the following program: 70% to 100% B in 4.5 min, held at 100% B for 2 min, re-equilibrated at 70% B for 4 min. The investigated metabolites along with their specific MRM transitions, MS/MS parameters, and its ionic chromatography are shown in **Table S4**.

### Animal Study

#### MZRW Treatment in Oleamide-Induced Slow Intestinal Motility in Mice

Male C57BL/6J mice weighing 22-25 g were purchased from the Laboratory Animal Services Center, The University of Hong Kong. The animals were fed a normal rodent diet ad libitum with free access to water, and were housed in rooms maintained at 22 ± 1°C with a 12 h light/dark cycle (lights on 6:00-18:00). 32 mice were equally divided into 4 groups. Oleamide-induced slow intestinal motility model in mice was similar to that as in a previous publication (Capasso et al., 2005). In brief, mice were intraperitoneally (i.p.) injected with blank solution (5% EtOH), or oleamide (Sigma-Aldrich, United States) (10 mg/kg, dissolved in 5% EtOH) 30 min prior to drug treatment. During treatment, mice in the control and oleamide-treatment groups received saline water orally, one oleamide-treating group, low- (10 g/kg) and high dose (20 g/kg) MZRW were administrated *via* lavage. Fecal pellet number was counted every 30 minutes after oleamide administration for 2h.

#### MZRW Treatment in Normal Mice

Male C57BL/6J mice weighing 22-25 g were purchased from the Laboratory Animal Services Center, The Chinese University of Hong Kong. 23 mice were divided into two groups: MZRW (n = 12) and saline control (n = 11). The mice received an oral administration of either MZRW (10 g/kg) or saline, respectively. Every two mice were assigned a cage and their cumulative fecal pellet number recorded after 60 min and 120 min. Mice were sacrificed after the experiment, with serum, ileum and colon samples collected and immediately stored at -80 °C.

#### RNA Extraction and Quantitative PCR Analysis

The ileum and colon samples (30 mg) were weighed and homogenized with TissueLyzer (Qiagen, Germany) and total RNA was isolated and extracted by TRIzol protocol (Thermo Fisher scientific, US) (Lin et al., 2015). Assays were performed to evaluate the expression of target genes using a 7900HT Fast Real-time PCR system (Applied Biosystems, Life Technologies, California, USA). The primer sequences for FAAH were 5’-GTGGTGCTRACCCCCATGCTGG-3’ (forward) and 5’-TCCACCTCCCGCATGAACCGCAGACA-3’ (reverse) (Capasso et al., 2005). mRNA expression was calculated using the ΔΔ-C_T_ analysis method (Pfaffl, 2001), with β-actin levels used as an internal reference.

### Human Ethics

The human study protocol was approved by the Ethics Committee on the Use of Human Subjects for Teaching and Research, Hong Kong Baptist University (Approval no HASC/10-11/16) in September 2011. It was registered at ClinicalTrials.gov (NCT01695850) in September 2012, and published in 2013 (Zhong et al., 2013).

### Animal Ethics

The Animal Ethics Committee of Hong Kong Baptist University approved all experimental protocols in accordance with “Institutional Guidelines and Animal Ordinance” from Department of Health, Hong Kong Special Administrative Region (HASC/16-17/0331).

### Statistical Analysis

The principal component analysis (PCA) and correlation analysis were performed by using the *scikit-learn* module (Pedregosa et al., 2011). The Pearson correlation analysis was performed between metabolic alteration and CSBM improvement in the various treatment groups. Results were ranked by Pearson r and *p* values. Paired t-test, one-way ANOVA, and χ^2^ test were used to analyze the change of oleamide in quantitative analysis. Alterations of genes and proteins in mice were compared using the Student t-test. All statistical analyses were two-tailed, and the significance level was set to 0.05. Quantitative analyses of oleamide and animal test were done in Prism 6 (GraphPad Software Inc., United States).

## Results

### Change of Oleamide Levels Is Negatively Correlated With Improvement of CSBM in FC Patients Treated With MZRW

**Figure 1:**
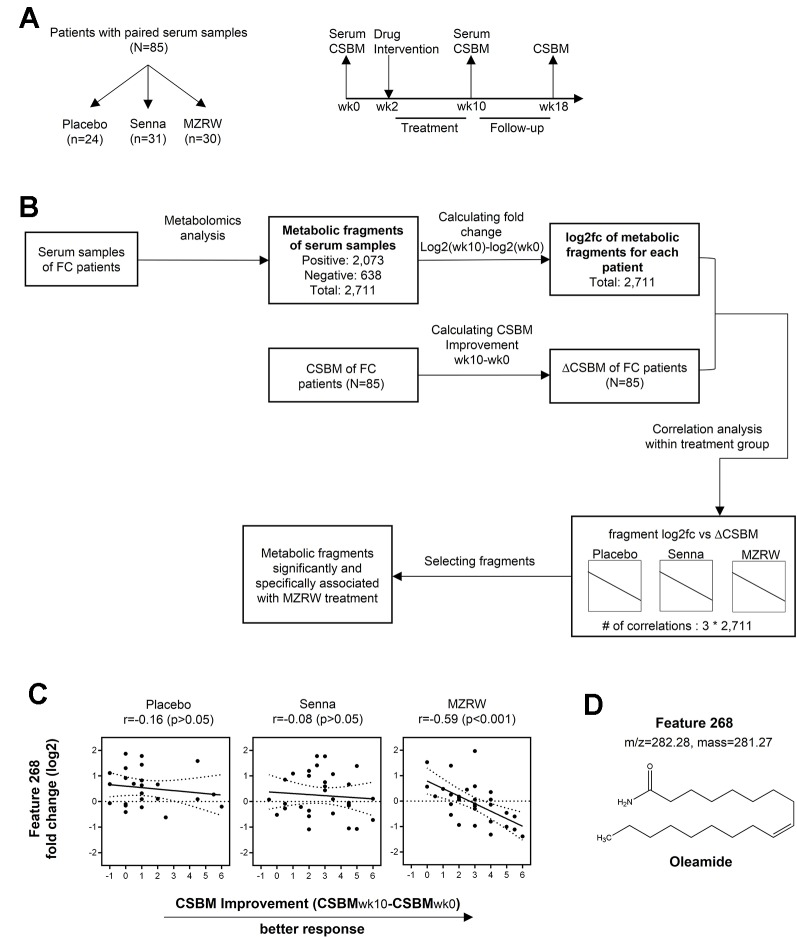
Serum pharmacometabolomics analysis of FC subjects from a three-armed, randomized, controlled clinical study. **(A)** Study design to investigate a metabolic biomarker associated with treatment efficacy in FC patients. Patients with paired serum samples were divided into three groups based on different treatment approaches: Placebo group (n = 24), Senna group (n = 31), and MZRW group (n = 30). For all patients, serum samples were acquired before and after treatment, at week 0 and week 10, respectively. Meanwhile, the number of complete spontaneous bowel movements per week (CSBM) for each patient were also recorded at the same time point, as well as at week 18. **(B)** The phamacometabolomic analysis flowchart for FC patients treated with different therapies. Metabolic features which are significantly and specifically associated with MZRW treatment were selected after calculations. **(C)** One metabolic feature (ID: 268) was found to be negatively correlated (Pearson r = -0.59, *p* = 0.00057) with change of CSBM (ΔCSBM) in the MZRW group, but not in the Senna or placebo group. **(D)** According to mass:charge ratio (m/z) and mass, feature 268 was identified as oleamide.

In the clinical study, to compare the efficacy of MZRW with that of Senna and a placebo, 291 FC patients were randomly assigned into three distinct groups (Zhong et al., 2019) for 8 weeks of treatment and 8 weeks of follow-up, as shown in the flowchart (**Figure 1A**). For pharmacometabolomic analysis, we randomly selected 30 subjects from each group, and collected serum samples at week 0 (pre-treatment or baseline) and week 10 (post-treatment). Some patients dropped out during the treatment period. At the end, paired serum samples from 85 subjects were collected: 30 from the MZRW group, 31 from the Senna group, and 24 from the placebo group. Subjects from the three treatment groups were of both gender, and of comparable age and body weight index (**Table 1**). During the treatment period, CSBM improvement in the MZRW group [2.9 (95%CI: 2.3, 3.5)] and the Senna group [2.8 (95%CI: 0.5, 2.1)] was similar, and both fared better than the placebo group [1.3 (95%CI: 0.5, 2.1)]. In the follow-up period, the CSBM improvement in the MZRW group [2.2 (95%CI: 1.5, 2.9)] was much better than that of the Senna [1.2 (95%CI: 0.7, 1.6)] and placebo group [0.5 (95%CI: 0.0, 1.0)]. These results on CSBM improvement were consistent with that observed in whole subjects (Zhong et al., 2019) and suggest that MZRW might have distinct pharmacological activity for FC, resulting in a more sustained efficacy during the follow-up period.

**Table 1:** Statistics of FC patients with paired serum samples at pre- and post-treatment.

Treatment	Patient #	Age (y)	BMI (kg/m^3^)	CSBM Improvement	
				wk10^a^	wk18^b^
MZRW	30 (M: 4; F: 26)^c^	44.5 (95%CI:41.0,48.0)	21.9 (95%CI:21.0,22.8)	2.9 (95%CI:2.3,3.5)	2.2 (95%CI:1.5,2.9)
Senna	31 (M: 2; F: 29)	46.9 (95%CI:43.2,50.7)	21.4 (95%CI:20.4,22.3)	2.8 (95%CI:2.2,3.4)	1.2 (95%CI:0.7,1.6)
Placebo	24 (M: 3; F: 21)	44.6 (95%CI:38.8,50.4)	21.7 (95%CI:20.7,22.7)	1.3 (95%CI:0.5,2.1)	0.5 (95%CI:0.0,1.0)

a CSBM improvement at wk10 in MZRW group and Senna are significantly higher than that in placebo group (*p* < 0.01), while there is no statistical difference between MZRW- and Senna group.

b CSBM improvement at wk18 in MZRW group is significantly higher than it in Senna (*p* < 0.05) and placebo (*p* < 0.001) groups.

c M, male; F, female.

To study post-treatment systematic changes, pharmacometabolomic analysis was conducted. Discovering which metabolites correlated with CSBM improvement in FC patients treated with MZRW, but not with Senna or a placebo, was of special interest. To investigate this, untargeted metabolomics analysis was performed for the paired serum samples of 85 subjects. In total, 2,073 fragments were found in positive mode, while 638 were found in negative mode. According to the multivariate statistical analysis, no obvious change of whole metabolic profile was observed between pre-treatment (baseline) and post-treatment samples (**Figure S1**).

The alteration of individual fragment was calculated as log2 fold change (log2fc) between the week 10 sample and week 0 sample from the same patient group (**Figure 1B**). Meanwhile, CSBM improvement of this patient group (CSBM_wk10_-CSBM_wk0_) was also calculated (**Figure 1B**). For each treatment group, the Pearson correlation analysis was performed to analyze the association between metabolite alteration and CSBM improvement for the 2,711 metabolic fragments found in both positive and negative modes (**Figure 1B**). The metabolites of which alteration significantly correlated (defined as *p* < 0.01) with CSBM improvement in the MZRW treatment group, but not significantly correlated with CSBM improvement in the Senna or placebo group, were selected (**Table S5**).

Among the 15 metabolic features which satisfied the aforementioned criteria, 5 were matched with endogenous metabolites in the Metabolite and Tandem MS Database (https://metlin.scripps.edu) (**Figure S2**). One of them, which was unambiguously matched to oleamide, drew our attention. It is the metabolic feature of which alteration is most negatively correlated with CSBM improvement in the MZRW group (Pearson *r* = -0.59, *p* = 0.00057), but unchanged in both the Senna and placebo groups (**Figures 1C**, **D**). Furthermore, two oleamide derivatives, oleoyl ethyl amide and N-oleoyl taurine, also correlated significantly with MZRW efficacy (**Figure S2**).

Oleamide is the amide derivative of oleic acid and belongs to the family of fatty acid amide (FAA). Oleamide is synthesized from N-Oleoylglycine by peptidylglycine α-amidating monoxygenase (PAM), and is degraded to oleic acid by fatty acid amide hydrolase (FAAH)(Hiley and Hoi, 2007). Compounds of the FAA family are known as endocannabinoids which bind the cannabinoid receptors. Activation of the cannabinoid receptor 1 (CB1) by oleamide inhibits cAMP production and Ca^2+^ influx (Fonseca et al., 2013). In particular, oleamide has been reported to cause slow intestinal motility in mice through activating the CB1 receptors (Capasso et al., 2005).

Taken together, alteration of oleamide, a known regulator of intestinal motility, is significantly and specifically correlated with CSBM improvement (therapeutic efficacy) of MZRW, suggesting that the oleamide signaling pathway could be targeted by MZRW to relieve FC.

### MZRW Treatment Down-Regulates Serum Oleamide Levels in FC Patients

**Figure 2:**
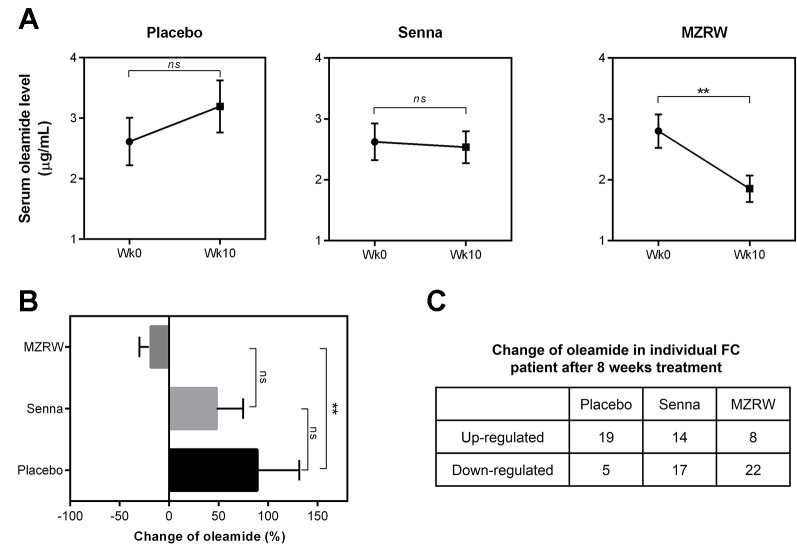
MZRW reduces circulating oleamide in FC patients. **(A)** Serum levels of oleamide pre-treatment (wk0) and post-treatment (wk10) in FC patients treated with Placebo, Senna, and MZRW. The differences at different time points were evaluated by paired, two-tailed Student t-test (ns, *p* > 0.05; **, *p* < 0.01). **(B)** Percentage of serum oleamide alterations in FC patients treated with Placebo, Senna, and MZRW. **(C)** Number of FC patients with up- or down-regulation of oleamide treated with Placebo, Senna, and MZRW.

To further confirm the role of MZRW in oleamide regulation, we quantitatively analyzed serum oleamide levels between pre- and post-treatment samples of FC patients. Interestingly, in the placebo group, the oleamide level is mildly increased [from 2.613 µg/mL (95%CI: 1.800, 3.425) to 3.195 µg/mL (95%CI: 2.305, 4.085)], but not statistically significant (*p* = 0.227) after an 8-week treatment (**Figure 2A**). The Senna group saw no changes in oleamide levels over 10 weeks (from 2.623 µg/mL (95%CI: 2.008, 3.239) to 2.536 µg/mL (95%CI, 1.999, 3.073), *p* = 0.824) (**Figure 2A**). However, in the MZRW group, serum oleamide levels were significantly reduced (from 2.800 µg/mL (95%CI: 2.236, 3.364) to 1.854 µg/mL (95%CI: 1.408, 2.299), *p* = 0.007) (**Figure 2A**). Relative change of oleamide in percentage during treatment period was also analyzed (placebo, +89.10% [95%CI: 1.30, 176.90); Senna, +47.94% (95%CI: -6.75, 102.60); MZRW, -18.61% (95%CI: -41.99, 4.77)] (**Figure 2B**). Consistently, oleamide was downregulated in 22 out of 30 subjects in the MZRW group; while oleamide downregulation was seen much less in subjects from the placebo and Senna groups (χ^2^ test, *p* = 0.0006, **Figure 2C**). Taken together, this quantitative analysis suggests that MZRW treatment causes down-regulation of circulating oleamide in FC patients.

### MZRW Rescues Oleamide-Induced Slow Intestinal Motility in Mice

**Figure 3:**
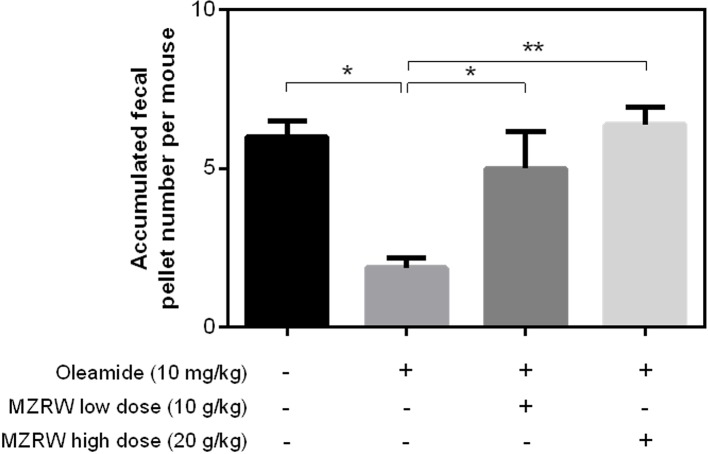
MZRW attenuates oleamide-induced slow GI motility in mice. Mice were equally divided into four groups (n = 8/group). The control group were intraperitoneally (i.p.) injected with blank solution (5% EtOH), while the remaining groups were pre-treated with oleamide (10 mg/kg in 5% EtOH), at 30 min before MZRW treatment. During treatment, the control group and oleamide-only group were orally administered with saline water, while the remaining two groups were administered with MZRW low dose (10 g/kg) and MZRW high dose (20 g/kg), respectively. From the beginning of drug treatment, the fecal pellet number of mice were recorded every 30 min in 2h. The accumulated fecal pellet number was calculated and the statistical differences evaluated by one-way ANOVA and Student t-test in Prism 6 (*, *p* < 0.05; **, *p* < 0.01).

Furthermore, we tested if MZRW could antagonize the slow intestinal motility induced by oleamide. Consistent with previous findings (Capasso et al., 2005), the fecal pellet number for 2h in mice injected (i.p.) with oleamide (10 mg/kg) is significantly lower than that of a buffer-treated control group (1.9 (95%CI: 0.9, 2.9) vs 6.0 (95%CI: 3.8, 8.2), p < 0.05) (**Figure 3**). For mice treated with MZRW after oleamide injection, the fecal pellet number is significantly increased (**Figure 3**). In particular, the fecal pellet number in mice treated with low dose MZRW (10 g/kg) is 5.0 (95%CI: 1.3, 8.7), whereas the number in mice treated with high dose MZRW (20 g/kg) is 6.4 (95%CI: 4.6, 8.1) – both are significantly higher than that of a saline group (p < 0.05 and p < 0.01, respectively). No significant differences were found between the saline-treated control group and the MZRW low-dose treatment or MZRW high-dose treatment groups (**Figure 3**). This data indicates that MZRW attenuates oleamide-induced slow intestinal motility in mice.

### MZRW Down-Regulates Oleamide Through Enhancing Colonic FAAH-Mediated Degradation

**Figure 4:**
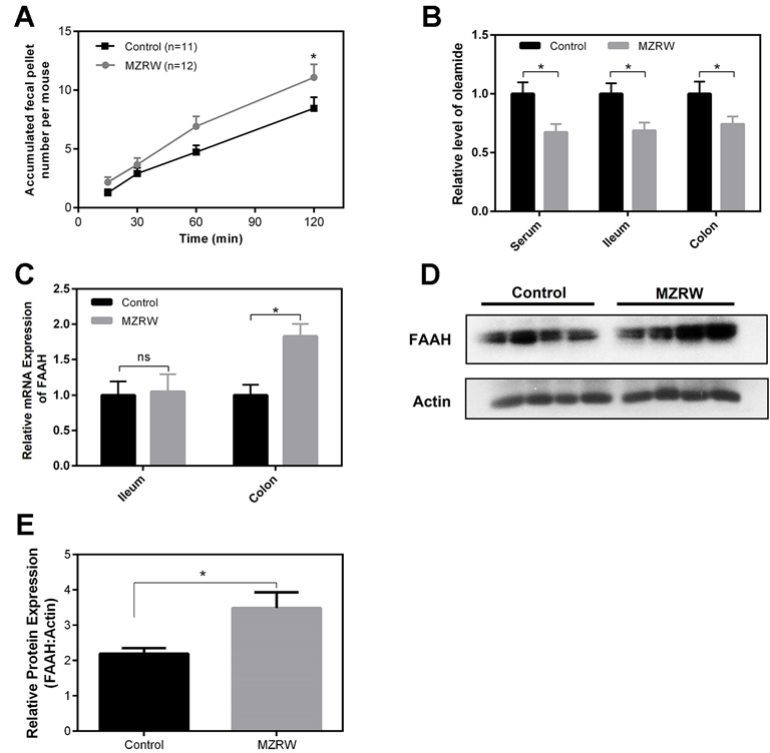
MZRW down-regulates oleamide through enhancing expression of FAAH in normal mice. **(A)** Mice were treated with MZRW and saline (control group). The fecal pellet number was recorded at 15, 30, 60, and 120 min. At each time point, the fecal pellet number in the MZRW and the control treatment group was compared with Student t-test (*, *p* < 0.05). **(B)** Oleamide levels in serum, ileum, and colon tissues in normal mice treated with saline (control) and MZRW. Two groups were compared using Student t-test (*, *p* < 0.05) **(C)** The relative mRNA expression of FAAH in ileum and colon tissues in normal mice treated by saline (control) and MZRW. The effect of MZRW treatment on mRNA expression was evaluated by Student t-test (ns, *p* > 0.05; *, *p* < 0.05). **(D)** The western blot results of FAAH in colonic tissues of mice treated with saline (control) and MZRW. **(E)** The relative protein expression level of FAAH in colonic tissues. Two groups were compared using Student t-test (*, *p* < 0.05).

Finally, we used normal mice to explore the possible mechanism by which MZRW regulates oleamide levels. In normal mice, MZRW increases colonic motility in terms of fecal pellet output (**Figure 4A**). Compared with saline (control) treatment, MZRW treatment (10 g/kg) reduces oleamide in serum, ileum, and colon samples of normal mice (**Figure 4B**). PCR analysis revealed the upregulation of mRNA and protein levels of FAAH, the key enzyme which is responsible for degradation of oleamide in colon tissues (**Figures 4C**, **D**). In particular, the relative protein level of FAAH (FAAH: β-actin ratio) in the control group was 2.19 ± 0.15, while it was 3.49 ± 0.45 (*p* = 0.035) in the MZRW group (**Figure 4E**). These results suggest that MZRW down-regulates oleamide, possibly through increased FAAH-mediated degradation.

## Discussion

Network pharmacology has been successfully applied to explain the mechanisms-of-action of Chinese herbal medicine (Lu et al., 2004; Lu et al., 2012; Mohd Fauzi et al., 2013; Huang et al., 2014). This methodology, however, is generally “bottom-up”: the pharmacology of herbal medicine is regarded as the sum of individual contributions from many compounds. Our previous work with network pharmacology identified five major active compounds from MZRW which can stimulate the contractions of colonic smooth muscle through multiple signaling pathways (Huang et al., 2018). In this work, we utilized pharmacometabolomics, which is “top-down”, to uncover the distinct pharmacology of MZRW. We successfully identified oleamide, the metabolite for which alterations are significantly and specifically correlated with the efficacy of MZRW.

As one member of the FAA family, oleamide has been known for its role in the regulation of intestinal motility in mice by activating the CB1 receptor (Capasso et al., 2005). Nevertheless, the role of oleamide in human FC patients is still largely unknown. Interestingly, our study showed that the serum oleamide levels are likely to be increased in FC patients treated with a placebo over 10 weeks. In line with our observation, the decreased expression of FAAH was found in the intestinal tissues of slow-transit constipation patients (Qiao et al., 2010; Zhang et al., 2014), suggesting that oleamide signaling might be implicated in the progress of FC. This hypothesis could be better validated in an independent longitudinal study.

We found that MZRW treatment decreases the oleamide levels in FC patients, which explains the unique pharmacology of MZRW compared with Senna. Animal studies demonstrated the regulation of oleamide by MZRW possibly through augmentation of FAAH-mediated degradation. Bashashati et al. proposed that inhibiting endocannabinoid biosynthesis could be a novel approach for constipation treatment, which has been validated by animal studies (Bashashati et al., 2012; Bashashati et al., 2015). Very interestingly, our findings suggest a novel FC treatment approach: enhancing endocannabinoid degradation by promoting the expression/activity of intestinal FAAH. Further preclinical and clinical studies are required to prove such a hypothesis.

To test if patients with lower oleamide respond better to MZRW therapy, we performed correlation analysis between baseline serum oleamide level and the CSBM improvement in week 10 (**Figure S3**). In the MZRW group, a negative correlation trend was observed (r = -0.16, *p* = 0.40), but it was not statistically significant. In contrast, no positive/negative correlation was observed in either the Senna (r = -0.04, *p* = 0.83) or placebo (r = 0.06, *p* = 0.76) group. It is still possible that patients with lower oleamide are better responders to MZRW. However, such a hypothesis may be validated in another cohort study with a larger sample size.

In mice treated with MZRW, the levels of oleamide are decreased in both ileum and colon, but the FAAH level was only up-regulated in colon. An explanation for these phenomena is that the major site of MZRW action on FAAH could be in the colon, but not the ileum. Our pharmacokinetics (PK) study of MZRW in rats (Huang et al., 2018) found that a number of compounds of MZRW can be identified in feces, suggesting that the colon could be a major site for absorption of MZRW-active compounds. Enhanced degradation of oleamide in the colon decreases oleamide levels in circulation. Finally, oleamide in ileum may be down-regulated as a consequence of decreased oleamide in circulation.

A limitation of this study is that the active compounds in MZRW which are responsible for the regulation of oleamide have not been identified. A cell-based assay, which can detect FAAH change during drug treatment, has been established in the author’s lab, with screening work underway. We also cannot exclude the possibility that some compounds from MZRW could enhance the transportation of oleamide. Shuttling the endocannabinoids from the plasma membrane to intracellular targets such as FAAH, is an important process of endocannabinoid degradation. Fatty-acid-binding proteins (FABPs) and heat shock protein 70 (HSP70) are known transporter proteins of endocannabinoid (Fowler, 2013). It could be possible that active compounds from MZRW also upregulate the expression of these transporter proteins, thereby accelerating the degradation of oleamide.

In conclusion, a pharmacometabolomic approach was applied to investigate the clinical pharmacology of MZRW for FC. Serum oleamide was found to be negatively correlated with the efficacy of MZRW, but not with that of Senna or a placebo. The causal link between MZRW treatment and down-regulation of oleamide has been validated by quantitative analysis. In the animal study, MZRW improves bowel movement through down-regulating oleamide, possibly by enhancing the FAAH-mediated oleamide degradation. Wisdom from MZRW, a 2,000 year old Chinese Herbal Medicine, sheds light on the way to develop novel therapies for FC.

## Data Availability Statement

All datasets generated for this study are included in the article/**Supplementary Material**.

## Ethics Statement

The studies involving human participants were reviewed and approved by The Ethics Committee on the Use of Human Subjects for Teaching and Research, Hong Kong Baptist University. The patients/participants provided their written informed consent to participate in this study. The animal study was reviewed and approved by The Animal Ethics Committee of Hong Kong Baptist University.

## Author Contributions

Z-XB is acting as guarantor of this article. Z-XB and TH designed the whole study. TH, LZha, and C-YL performed pharmacometabolomic analysis. LZha carried out quantitative analysis of oleamide of all biological samples. LZha, C-YL, LL, and Z-WN did animal testing. D-DH performed identification of chemical compositions of MZRW granules and extract. Z-XB, TH, LZha, and C-YL analyzed the data and wrote the manuscript. LZho and Z-JY made contributions to the manuscript preparation. All authors reviewed and approved the final manuscript.

## Funding

This study was supported by the Health and Health Services Fund, Hong Kong SAR, P. R. China (project no. 09101501) and Shenzhen Science and Technology Innovations Committee (No. JCYJ20140419130444178). The sponsors have no roles in study design, collection, analysis, and interpretation of the data and in the writing of the manuscript.

## Conflict of Interest

The authors declare that the research was conducted in the absence of any commercial or financial relationships that could be construed as a potential conflict of interest.
