# Supplementary material for: Chinese Herbal Medicine (MaZiRenWan) Improves Bowel Movement in Functional Constipation Through Down-Regulating Oleamide
Source: Front Pharmacol. 2020 Jan 23;10:1570. doi: 10.3389/fphar.2019.01570 (PMC6989537; doi:10.3389/fphar.2019.01570)
Supplement: Supplementary file 1 [file DataSheet_1.docx]

Supplementary Material

# Supplementary Figures and Tables

## Supplementary Figures

**Supplementary Figure 1. Principle component analysis (PCA) of serum metabolomic features for FC patients under different approaches: MZRW (blue), Senna (orange), and Placebo (black). (a)** PCA analysis for metabolic features detected in positive mode. **(b)** PCA analysis for metabolic features detected in negative mode. In both modes, the patients samples acquired at pre-treatment phase are represented by open markers, while patients samples acquired at pre-treatment phase are represented by close markers. The responder is defined as patient whose improvement of CSBM (wk10-wk0) was greater than 1.

**Supplementary Figure 2. Structures of identified metabolites of which alterations are significantly correlated with the Complete Spontaneous Bowel Movement (CSBM) improvement of Functional Constipation patients treated by MZRW.** **(a)** Metabolic features identified in positive mode: 1, oleamide; 2, decanamide; 3, 2,4-diaminobutryic acid; 4, oleoyl ethyl amide. **(b)** Metabolic features identified in negative mode: 5, N-oleoyl taurine.


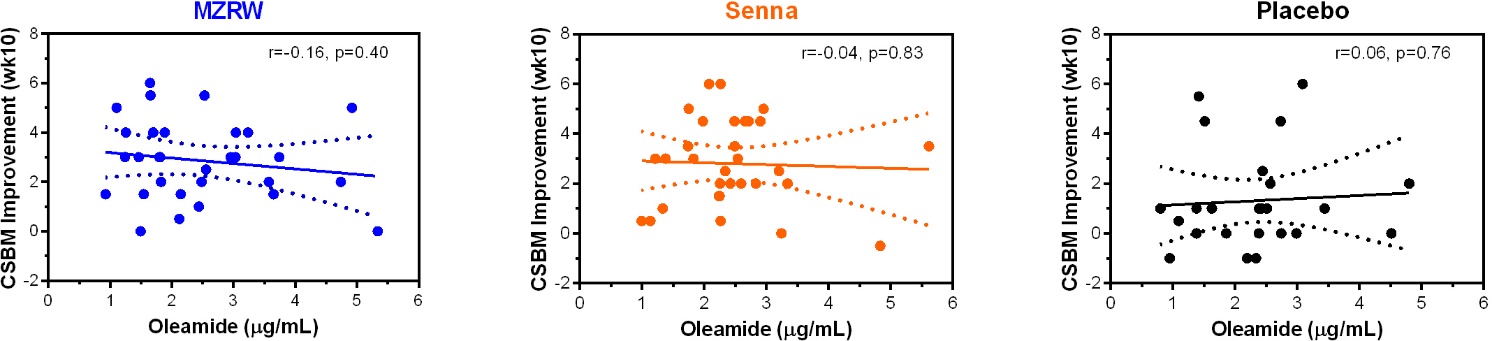


**Supplementary Figure 3. Correlation analysis between baseline serum oleamide and therapeutic efficacy in MZRW, Senna, and placebo group.** The therapeutic efficacy was represented by patient CSBM improvement at week 10 (wk10). The Pearson correlation coefficient (r) and corresponding p value were shown in the right upper corner in each panel.

## Supplementary Tables

**Supplementary Table 1. The individual specimens of MZRW**

| **Herb Ingredient** | **中文名** | **Plant Source** | **Medicinal parts** | **Batch number**  **(by PuraPharma)** | **Voucher number** |
| --- | --- | --- | --- | --- | --- |
| *Cannabis Fructus*  *(CF)* | 火麻仁 | *Cannabis sativa* L. | Dry seeds | RM140324-08 | HKBU-20140515-HMR |
| *Rhei Radix et Rhizoma*  (*RRR*) | 大黃 | *Rheum tanguticum* Maxim,ex Balf. | Dry roots | RM131122-07 | HKBU-20140515-DH |
| *Armeniacae Semen Amarum*  *(ASA)* | 苦杏仁 | *Prunus mandshurica* (Maxim.) Koehne | Drug seeds | RM140512-06 | HKBU-20140515-KXR |
| *Paeoniae Radix Alba*  *(PRA)* | 白芍 | *Paeonia lactiflora* Pall. | Dry roots | RM140423-08 | HKBU-20140515-BS |
| *Magnoliae Officinalis Cortex*  *(MOC)* | 厚樸 | *Magnolia officinalis* Rehder & E.H.Wilson | Dry barks | RM131231-11 | HKBU-20140515-HP |
| *Aurantii Fructus Immaturus*  *(AFI)* | 枳實 | *Citrus aurantium* L. | Dry fruit | RM140603-06 | HKBU-20140515-ZS |

**Supplementary Table 2. Chemical profile of MZRW granules identified by UPLC-Q-TOF-MS**

| **Peak No.** | **RT** | **Observed mass** | **Ion species** | **Molecular formula** | **Error (mDa)** | **Identification** |
| --- | --- | --- | --- | --- | --- | --- |
| 1 | 0.735 | 225.0614 | [M + HCOO]- | C6 H12 O6 | -1.12 | Inositol |
| 2 | 1.61 | 169.0133 | [M - H]- | C7 H6 O5 | -0.93 | Gallic acid |
| 3 | 1.61 | 331.0659 | [M - H]- | C13 H16 O10 | 1.52 | Gallic acid 3-O-β-D-glucopyranoside |
| 4 | 1.634 | 125.0253 | [M - H]- | C6 H6 O3 | 0.89 | 1,2,3-benzenetriol |
| 5 | 1.984 | 331.066 | [M - H]- | C13 H16 O10 | 1.09 | Gallic acid 4-O-β-D-glucopyranoside |
| 6 | 2.588 | 493.1199 | [M - H]- | C19 H26 O15 | 0.1 | 6-O-galloylsucrose |
| 7 | 3.871 | 451.1234 | [M - H]- | C21 H24 O11 | -1.41 | Hesperetin-7-O-β-D-glucoside |
| 8 | 4.509 | 451.1234 | [M + HCOO]- | C20 H22 O9 | -1.32 | Piceatannol 3-O-β-D-glucopyranoside |
| 9 | 4.661 | 577.1335 | [M - H]- | C30 H26 O12 | -1.72 | Procyanidin B-1 |
| 10 | 4.852 | 451.1235 | [M - H]- | C21 H24 O11 | -2.82 | (+)-catechin-5-O-β-D-glucopyranoside |
| 11 | 4.956 | 289.071 | [M - H]- | C15 H14 O6 | -0.87 | (+)-catechin |
| 12 | 4.956 | 483.0767 | [M - H]- | C20 H20 O14 | -0.54 | 1,6-di-O-galloylycerol-β-D-glucopyranoside |
| 13 | 5.268 | 417.1387 | [M + HCOO]- | C17 H24 O9 | -1.46 | Paeonin A |
| 14 | 5.528 | 456.1511 | [M - H]- | C20 H27 N O11 | 0.47 | Amygdalin |
| 15 | 5.828 | 289.0711 | [M + HCOO]- | C14 H12 O4 | -0.63 | Piceatannol |
| 16 | 5.979 | 729.1466 | [M - H]- | C37 H30 O16 | 0.61 | Procyanidin B-1-3-O-gallate |
| 17 | 6.131 | 340.1037 | [M + HCOO]- | C14 H17 N O6 | -0.13 | Prunasin |
| 18 | 6.51 | 609.1829 | [M - H]- | C28 H34 O15 | 7.36 | Neohesperidin |
| 19 | 6.51 | 623.2 | [M - H]- | C29 H36 O15 | 1.71 | Acteoside |
| 20 | 6.541 | 449.1467 | [M + HCOO]- | C21 H24 O8 | 1.23 | Desoxyrhaponticin |
| 21 | 6.541 | 479.1565 | [M - H]- | C23 H28 O11 | 2.54 | Albiflorin |
| 22 | 6.541 | 525.1636 | [M - H]- | C24 H30 O13 | 2.1 | Mudanpioside E |
| 23 | 6.541 | 609.1843 | [M + HCOO]- | C27 H32 O13 | 2.54 | 10R-chrysaloin1-O-β-D-glucopyranoside |
| 24 | 6.554 | 119.0503 | [M - H]- | C8H8O | 0.01 | Benzeneacetaldehyde |
| 25 | 6.744 | 639.1921 | [M + HCOO]- | C28 H34 O14 | -0.15 | Poncirin |
| 26 | 6.962 | 567.2092 | [M - H]- | C27H36O13 | 0.81 | Citrusin B |
| 27 | 7.02 | 269.0448 | [M - H]- | C15 H10 O5 | -0.73 | Apigenin |
| 28 | 7.02 | 863.2018 | [2M - H]- | C21 H20 O10 | -0.26 | Emodin-8-O-β-D-glucopyranoside |
| 29 | 7.081 | 441.0817 | [M - H]- | C22 H18 O10 | -1.04 | (-)-Epicatechin-3-O-gallate |
| 30 | 7.081 | 445.077 | [M - H]- | C21 H18 O11 | -0.48 | Rhein-8-O-β-D-glucopyranoside |
| 31 | 7.142 | 417.1184 | [M - H]- | C21 H22 O9 | -5.11 | Cassialoin |
| 32 | 7.172 | 477.1395 | [M - H]- | C23 H26 O11 | -0.97 | Lindleyin/isolindleyin |
| 33 | 7.264 | 525.1606 | [M + HCOO]- | C23 H28 O11 | -3.17 | Paeoniflorin |
| 34 | 7.35 | 623.1973 | [M - H]- | C29 H36 O15 | -1.03 | Physcion-8-O-β-D-gentiobioside |
| 35 | 7.411 | 631.1659 | [M - H]- | C30 H32 O15 | 1.6 | Albiflorin; 4-O-(3,4,5-Trihydroxybenzoyl) |
| 36 | 7.597 | 609.1816 | [M + HCOO]- | C27 H32 O13 | -0.6 | Cascaroside C |
| 37 | 7.668 | 861.1833 | [M - H]- | C42H38O20 | 1.22 | Sennoside A or Sennoside B |
| 38 | 7.696 | 579.1717 | [M - H]- | C27 H32 O14 | 0 | Naringin |
| 39 | 7.935 | 298.1075 | [M - H]- | C17 H17 N O4 | -1.65 | N-trans-caffeoyltyramine |
| 40 | 8.026 | 355.1022 | [M + HCOO]- | C15 H18 O7 | -1.55 | 2-O-cinnamoyl-β-D-glucose |
| 41 | 8.13 | 609.1823 | [M + HCOO]- | C27 H32 O13 | -0.22 | Cascaroside D |
| 42 | 8.13 | 609.1824 | [M - H]- | C28 H34 O15 | -0.49 | Hesperidin |
| 43 | 8.398 | 189.0546 | [M - H]- | C11 H10 O3 | -1.06 | 2,5-dimethyl-7-hydroxychromone |
| 44 | 8.398 | 393.1182 | [M - H]- | C19 H22 O9 | -1.43 | Aloesone-7-O-β-D-glucopyranoside |
| 45 | 8.428 | 431.0975 | [M - H]- | C21 H20 O10 | -0.76 | Kaempferol-3-O-rhamnoside |
| 46 | 8.428 | 525.1595 | [M + HCOO]- | C23 H28 O11 | -2.09 | Mudanpioside I |
| 47 | 8.652 | 593.1917 | [M - H]- | C34H30N2O8 | -0.62 | Cannabisin A |
| 48 | 8.667 | 461.1086 | [M + HCOO]- | C21 H20 O9 | -0.23 | Chrysophaein |
| 49 | 8.778 | 595.2073 | [M - H]- | C34 H32 N2 O8 | -1.36 | Cannabisin B |
| 50 | 8.877 | 315.1232 | [M - H]- | C18 H20 O5 | -0.61 | Honokitriol; (7R*,8R*)-form |
| 51 | 9.615 | 461.1082 | [M - H]- | C22 H22 O11 | -0.47 | 1-O-galloyl-6-O-cinnamoyl-β-D-glucose |
| 52 | 9.645 | 639.1922 | [M + HCOO]- | C28 H34 O14 | -0.94 | Neoponcirin |
| 53 | 9.798 | 415.1019 | [M - H]- | C21 H20 O9 | 0.47 | Chrysophanol-8-O-β-D-glucopyranoside |
| 54 | 9.872 | 847.2097 | [M - H]- | C42 H40 O19 | 4.51 | Sennoside C |
| 55 | 9.949 | 313.0344 | [M - H]- | C16 H10 O7 | -1 | laccaic acid D |
| 56 | 10.065 | 253.0496 | [M - H]- | C15 H10 O4 | -1.01 | Chrysophanol |
| 57 | 10.065 | 831.2135 | [2M - H]- | C21 H20 O9 | -0.43 | Chrysophanol-1-O-β-D-glucopyranoside |
| 58 | 10.385 | 517.0975 | [M - H]- | C24 H22 O13 | -1.16 | Emodin-8-O-(6'-O-malonyl)-glucoside/7-O-glucosyl-6''-malonyl genistein |
| 59 | 10.39 | 299.1281 | [M - H]- | C18 H20 O4 | -0.86 | Magnolignan A |
| 60 | 10.449 | 629.1871 | [M + HCOO]- | C30 H32 O12 | -0.29 | 6'-O-galloylalbiflorin |
| 61 | 10.449 | 629.1871 | [M - H]- | C31 H34 O14 | -0.37 | Mudanpioside B |
| 62 | 10.613 | 629.187 | [M + HCOO]- | C30 H32 O12 | -1.03 | Benzoylpaeoniflorin |
| 63 | 10.707 | 723.2134 | [M - H]- | C33 H40 O18 | -0.51 | Melitidin |
| 64 | 10.745 | 285.0396 | [M + HCOO]- | C14 H8 O4 | -0.78 | 1,8-Dihydroxyanthraquinone |
| 65 | 10.745 | 285.0395 | [M - H]- | C15 H10 O6 | -1.05 | Citreorosein |
| 66 | 10.916 | 283.0607 | [M - H]- | C16 H12 O5 | -0.47 | Physcion |
| 67 | 10.916 | 445.113 | [M - H]- | C22 H22 O10 | -1.18 | Physcion-1-O-β-D-glucopyranoside |
| 68 | 11.05 | 241.0858 | [M - H]- | C15 H14 O3 | -1.28 | Magnatriol B |
| 69 | 11.7 | 297.0396 | [M - H]- | C16 H10 O6 | -0.9 | 6-methyl-rhein |
| 70 | 12.133 | 253.0865 | [M - H]- | C16 H14 O3 | -0.65 | Magnaldehyde D |
| 71 | 12.193 | 515.1925 | [M + HCOO]- | C26H30O8 | 0.11 | Limonin |
| 72 | 12.245 | 283.0242 | [M - H]- | C15 H8 O6 | -0.49 | Rhein |
| 73 | 12.558 | 269.0813 | [M - H]- | C16 H14 O4 | -0.66 | Imperatorin |
| 74 | 12.59 | 589.1344 | [2M + HCOO]- | C15 H12 O5 | -0.68 | Naringenin |
| 75 | 13.379 | 269.045 | [M - H]- | C15 H10 O5 | -0.64 | Emodine |
| 76 | 13.809 | 265.1243 | [M - H]- | C18H18O2 | 0.81 | Honokiol |
| 77 | 13.991 | 281.1182 | [M - H]- | C18 H18 O3 | -0.19 | Obovatol |
| 78 | 14.055 | 265.1247 | [M - H]- | C18 H18 O2 | 1.25 | Magnolol |
| 79 | 16.084 | 327.2535 | [M + HCOO]- | C18H34O2 | -0.64 | Oleic acid |
| 80 | 22.057 | 279.223 | [M - H]- | C18H32O2 | 0.08 | Linoeic acid |

**Supplementary Table 3. Chemical profile of MZRW extract identified by UPLC-Q-TOF-MS**

| **Peak No.** | **RT** | **Observed mass** | **Ion species** | **Molecular formula** | **Error (mDa)** | **Identification** |
| --- | --- | --- | --- | --- | --- | --- |
| 1 | 0.704 | 195.0504 | [M + HCOO]^-^ | C_5_H_10_O_5_ | -0.65 | 2,3,4-Trihydroxy-2-methylbutanoic acid;(2R,3R)-form |
| 2 | 0.735 | 225.0614 | [M + HCOO]^-^ | C_6_H_12_O_6_ | -1.12 | Inositol |
| 3 | 1.61 | 169.0133 | [M - H]^-^ | C_7_H_6_O_5_ | -0.93 | Gallic acid |
| 4 | 1.611 | 331.0659 | [M - H]^-^ | C_13_H_16_O_10_ | 1.52 | Mono-galloyl glucose isomer |
| 5 | 1.634 | 125.0253 | [M - H]^-^ | C_6_H_6_O_3_ | 0.89 | 1,2,3-benzenetriol |
| 6 | 1.984 | 331.066 | [M - H]^-^ | C_13_H_16_O_10_ | 1.09 | Mono-galloyl glucose isomer |
| 7 | 2.354 | 493.1185 | [M - H]^-^ | C_19_H_26_O_15_ | -1.23 | Galloylsucrose isomer |
| 8 | 2.588 | 493.1199 | [M - H]^-^ | C_19_H_26_O_15_ | 0.1 | Galloylsucrose isomer |
| 9 | 2.925 | 243.0499 | [M - H]^-^ | C_10_H_12_O_7_ | -1.06 | Glycerin-mono gallate |
| 10 | 3.871 | 451.1234 | [M + HCOO]^-^ | C_20_H_22_O_9_ | -0.89 | Oxyresveratrol 2-O-β-D-glucopyranoside |
| 11 | 4.57 | 475.1446 | [M - H]^-^ | C_20_H_28_O_13_ | -1.25 | Phloroacetophenone 4-neohesperidoside/isomer |
| 12 | 4.661 | 577.1335 | [M - H]^-^ | C_30_H_26_O_12_ | -1.72 | Procyanidin B-1 |
| 13 | 4.691 | 543.1174 | [M - H]^-^ | C_23_H_28_O_13_S | -0.49 | Paeoniflorin sulfonate I |
| 14 | 4.956 | 289.071 | [M - H]^-^ | C_15_H_14_O_6_ | -0.87 | (+)-catechin |
| 15 | 4.956 | 483.0767 | [M - H]^-^ | C_20_H_20_O_14_ | -0.54 | 2,6-di-O-galloyglucose |
| 16 | 5.268 | 417.1387 | [M + HCOO]^-^ | C_17_H_24_O_9_ | -1.46 | Paeonin A |
| 17 | 5.298 | 151.0398 | [M - H]^-^ | C_8_H_8_O_3_ | -0.77 | Methyl salicylate |
| 18 | 5.528 | 456.1511 | [M - H]^-^ | C_20_H_27_NO_11_ | 0.47 | Amygdalin |
| 19 | 5.828 | 289.0711 | [M + HCOO]^-^ | C_14_H_12_O_4_ | -0.63 | Piceatannol |
| 20 | 5.901 | 785.2514 | [M - H]^-^ | C_35_H_46_O_20_ | 0.38 | Echinacoside |
| 21 | 5.979 | 593.1503 | [M - H]^-^ | C_27_H_30_O_15_ | -0.22 | 3,8-di-C-glucosylapigenin |
| 22 | 5.979 | 729.1466 | [M - H]^-^ | C_37_H_30_O_16_ | 0.61 | Procyanidin B-1-3-O-gallate |
| 23 | 6.039 | 771.2342 | [M - H]^-^ | C_34_H_44_O_20_ | -0.83 | Glucosyl hesperidin/isomer |
| 24 | 6.131 | 340.1037 | [M + HCOO]^-^ | C_14_H_17_NO_6_ | -0.13 | Prunasin |
| 25 | 6.325 | 729.1458 | [M - H]^-^ | C_37_H_30_O_16_ | -0.64 | Procyanidin B-2-3-β-O-gallate |
| 26 | 6.511 | 623.2 | [M - H]^-^ | C_29_H_36_O_15_ | 1.71 | Acteoside |
| 27 | 6.541 | 449.1467 | [M + HCOO]^-^ | C_21_H_24_O_8_ | 1.23 | Desoxyrhaponticin |
| 28 | 6.541 | 525.1616 | [M + HCOO]^-^ | C_23_H_28_O_11_ | 2.54 | Albiflorin |
| 29 | 6.554 | 119.0503 | [M - H]^-^ | C_8_H_8_O | 0.01 | Benzeneacetaldehyde |
| 30 | 6.744 | 639.1921 | [M + HCOO]^-^ | C_28_H_34_O_14_ | -0.15 | Poncirin |
| 31 | 6.805 | 787.2675 | [M + HCOO]^-^ | C_34_H_46_O_18_ | -1.28 | Liriodendrin |
| 32 | 6.962 | 567.2092 | [M - H]^-^ | C_27_H_36_O_13_ | 0.81 | Citrusin B |
| 33 | 7.02 | 269.0448 | [M - H]^-^ | C_15_H_10_O_5_ | -0.73 | Apigenin |
| 34 | 7.02 | 863.2018 | [2M - H]^-^ | C_21_H_20_O_10_ | -0.26 | Aloe-emodin-8-monoglucoside |
| 35 | 7.081 | 441.0817 | [M - H]^-^ | C_22_H_18_O_10_ | -1.04 | (-)-Epicatechin-3-O-gallate |
| 36 | 7.081 | 445.077 | [M - H]^-^ | C_21_H_18_O_11_ | -0.48 | Rhein-8-monoglucoside |
| 37 | 7.142 | 417.1184 | [M + HCOO]^-^ | C_20_H_20_O_7_ | -0.78 | 5,6,7,8,4'-pentamethoxy flavone |
| 38 | 7.172 | 477.1395 | [M - H]^-^ | C_23_H_26_O_11_ | -0.97 | Lindleyin/isolindleyin |
| 39 | 7.264 | 525.1606 | [M + HCOO]^-^ | C_23_H_28_O_11_ | -3.17 | Paeoniflorin |
| 40 | 7.35 | 623.1973 | [M - H]^-^ | C_29_H_36_O_15_ | -1.03 | Physcion-8-O-β-D-gentiobioside |
| 41 | 7.411 | 631.1659 | [M - H]^-^ | C_30_H_32_O_15_ | 1.6 | Albiflorin; 4-O-(3,4,5-Trihydroxybenzoyl) |
| 42 | 7.597 | 609.1816 | [M + HCOO]^-^ | C_27_H_32_O_13_ | -0.6 | Cascaroside C |
| 43 | 7.668 | 861.1833 | [M - H]^-^ | C_42_H_38_O_20_ | 1.22 | Sennoside A or Sennoside B |
| 44 | 7.696 | 579.1717 | [M - H]^-^ | C_27_H_32_O_14_ | 0 | Naringin |
| 45 | 7.757 | 631.1661 | [M - H]^-^ | C_30_H_32_O_15_ | 2.75 | Galloylalbiroin |
| 46 | 7.874 | 631.1657 | [M - H]^-^ | C_30_H_32_O_15_ | -2.3 | Galloylpaeoniflorin/galloylalbiflorin |
| 47 | 7.935 | 298.1075 | [M - H]^-^ | C_17_H_17_NO_4_ | -1.65 | N-trans-caffeoyltyramine |
| 48 | 8.026 | 355.1022 | [M + HCOO]^-^ | C_15_H_18_O_7_ | -1.55 | 2-O-cinnamoyl-β-D-glucose |
| 49 | 8.13 | 609.1824 | [M - H]^-^ | C_28_H_34_O_15_ | -0.49 | Hesperidin |
| 50 | 8.13 | 631.1652 | [M - H]^-^ | C_30_H_32_O_15_ | -2.47 | Galloylpaeoniflorin/galloylalbiflorin |
| 51 | 8.221 | 651.1562 | [M - H]^-^ | C_29_H_32_O_17_ | -0.86 | 5,7,4’trihydroxy-8,3’-dimethoxyflavone-3-O-[3-hydroxy-3-methylglutaryl(1-6)] |
| 52 | 8.251 | 463.1239 | [M - H]^-^ | C_22_H_24_O_11_ | -0.26 | Hesperitin-glucoside |
| 53 | 8.252 | 609.1829 | [M - H]^-^ | C_28_H_34_O_15_ | 1.25 | Neohesperidin |
| 54 | 8.398 | 189.0546 | [M - H]^-^ | C_11_H_10_O_3_ | -1.06 | 2,5-dimethyl-7-hydroxychromone |
| 55 | 8.398 | 393.1182 | [M - H]^-^ | C_19_H_22_O_9_ | -1.43 | Aloesone-7-O-β-D-glucopyranoside |
| 56 | 8.428 | 431.0975 | [M - H]^-^ | C_21_H_20_O_10_ | -0.76 | Kaempferol-3-O-rhamnoside |
| 57 | 8.428 | 525.1595 | [M + HCOO]^-^ | C_23_H_28_O_11_ | -2.09 | Mudanpioside I |
| 58 | 8.652 | 593.1917 | [M - H]^-^ | C_34_H_30_N_2_O_8_ | -0.62 | Cannabisin A |
| 59 | 8.667 | 461.1086 | [M + HCOO]^-^ | C_21_H_20_O_9_ | -0.23 | Chrysophaein |
| 60 | 8.778 | 595.2073 | [M - H]^-^ | C_34_H_32_N_2_O_8_ | -1.36 | Cannabisin B |
| 61 | 8.877 | 315.1232 | [M - H]^-^ | C_18_H_20_O_5_ | -0.61 | Honokitriol; (7R*,8R*)-form |
| 62 | 9.025 | 312.1236 | [M - H]^-^ | C_18_H_19_NO_4_ | -0.51 | (E)-N-feruloyltyramine |
| 63 | 9.594 | 609.2221 | [M - H]^-^ | C_35_H_34_N_2_O_8_ | -2.01 | Cannabisin C |
| 64 | 9.615 | 461.1082 | [M - H]^-^ | C_22_H_22_O_11_ | -0.47 | 1-O-galloyl-6-O-cinnamoyl-β-D-glucose |
| 65 | 9.645 | 639.1922 | [M + HCOO]^-^ | C_28_H_34_O_14_ | -0.94 | Neoponcirin |
| 66 | 9.828 | 407.134 | [M - H]^-^ | C_20_H_24_O_9_ | -0.85 | Torachrysone 8-O-glucoside |
| 67 | 9.872 | 847.2097 | [M - H]^-^ | C_42_H_40_O_19_ | 4.51 | Sennoside C |
| 68 | 9.927 | 431.0979 | [M - H]^-^ | C_21_H_20_O_10_ | -0.54 | Isovitexin |
| 69 | 9.949 | 313.0344 | [M - H]^-^ | C_16_H_10_O_7_ | -1 | laccaic acid D |
| 70 | 10.065 | 253.0496 | [M - H]^-^ | C_15_H_10_O_4_ | -1.01 | Chrysophanol |
| 71 | 10.385 | 517.0975 | [M - H]^-^ | C_24_H_22_O_13_ | -1.16 | Emodin-8-O-(6'-O-malonyl)-glucoside/7-O-glucosyl-6''-malonyl genistein |
| 72 | 10.39 | 299.1281 | [M - H]^-^ | C_18_H_20_O_4_ | -0.86 | Magnolignan A |
| 73 | 10.449 | 629.1871 | [M + HCOO]^-^ | C_30_H_32_O_12_ | -0.29 | 6'-O-galloylalbiflorin |
| 74 | 10.613 | 629.187 | [M + HCOO]^-^ | C_30_H_32_O_12_ | -1.03 | Benzoylpaeoniflorin |
| 75 | 10.707 | 723.2134 | [M - H]^-^ | C_33_H_40_O_18_ | -0.51 | Melitidin |
| 76 | 10.745 | 285.0396 | [M + HCOO]^-^ | C_14_H_8_O_4_ | -0.78 | 1,8-Dihydroxyanthraquinone |
| 77 | 10.916 | 283.0607 | [M - H]^-^ | C_16_H_12_O_5_ | -0.47 | Physcion |
| 78 | 10.916 | 445.113 | [M - H]^-^ | C_22_H_22_O_10_ | -1.18 | Physcion monoglucoside |
| 79 | 11.05 | 241.0858 | [M - H]^-^ | C_15_H_14_O_3_ | -1.28 | Magnatriol B |
| 80 | 11.157 | 473.108 | [M - H]^-^ | C_23_H_22_O_11_ | -1.03 | 2-​Acetylemodin 8-​O-​β-​D-​glucoside |
| 81 | 11.7 | 297.0396 | [M - H]^-^ | C_16_H_10_O_6_ | -0.9 | 6-methyl-rhein |
| 82 | 12.08 | 269.0469 | [M - H]^-^ | C_15_H_10_O_5_ | 0.99 | Aloe emodin |
| 83 | 12.193 | 515.1925 | [M + HCOO]^-^ | C_26_H_30_O_8_ | 0.11 | Limonin |
| 84 | 12.245 | 283.0242 | [M - H]^-^ | C_15_H_8_O_6_ | -0.49 | Rhein |
| 85 | 12.558 | 269.0813 | [M - H]^-^ | C_16_H_14_O_4_ | -0.66 | Imperatorin |
| 86 | 12.59 | 589.1344 | [2M + HCOO]^-^ | C_15_H_12_O_5_ | -0.68 | Naringenin |
| 87 | 13.379 | 269.045 | [M - H]^-^ | C_15_H_10_O_5_ | -0.64 | Emodin |
| 88 | 13.809 | 265.1243 | [M - H]^-^ | C_18_H_18_O_2_ | 0.81 | Honokiol |
| 89 | 13.991 | 281.1182 | [M - H]^-^ | C_18_H_18_O_3_ | -0.19 | Obovatol |
| 90 | 14.055 | 265.1247 | [M - H]^-^ | C_18_H_18_O_2_ | 1.25 | Magnolol |
| 91 | 15.04 | 357.2079 | [M - H]^-^ | C_22_H_30_O_4_ | 0.55 | Cannabichromenic acid |
| 92 | 16.084 | 327.2535 | [M + HCOO]^-^ | C_18_H_34_O_2_ | -0.64 | Oleic acid |
| 93 | 22.057 | 279.223 | [M - H]^-^ | C_18_H_32_O_2_ | 0.08 | Linoeic acid |

**Supplementary Table 4. The MRM transitions and MS/MS parameters of oleamide and AEA-d_8_**

| Name | Prec Ion | Prod Ion | Frag (V) | CE (V) | Polarity |
| --- | --- | --- | --- | --- | --- |
| AEA-d_8_ | 356.3 | 63.1 | 120 | 16 | positive |
| Oleamide | 282.2 | 247.4 | 120 | 10 | positive |

**Supplementary Table 5. Metabolic features significantly correlated with CSBM improvement in FC patients treated with MZRW**

| Mode | Feature ID | Mass (Da) | Identification with METLIN^a^ | Correlation coefficient^b^ | | |
| --- | --- | --- | --- | --- | --- | --- |
|  |  |  |  | MZRW | Senna | Placebo |
| Positive | 268 | 281.2707 | Oleamide  (CAS: 301-02-0) | -0.59  (5.7E-4) | -0.087  (6.4E-1) | -0.16  (4.5E-1) |
|  | 112 | 171.1609 | Decanamide  (CAS:2319-29-1) | -0.53  (2.7E-3) | -0.15  (4.3E-1) | -0.070  (7.4E-1) |
|  | 211 | 249.1693 | Extraneous hits | -0.50  (5.2E-3) | -0.069  (7.1E-1) | -0.033  (8.8E-1) |
|  | 39 | 118.0771 | 2,4-diaminobutyric acid  (CAS:1758-80-1) | -0.48  (6.7E-3) | -0.033  (8.6E-1) | -0.055  (8.0E-1) |
|  | 1423 | 719.9256 | No hits | -0.47  (9.1E-3) | -0.11  (5.6E-1) | -0.070  (7.5E-1) |
|  | 328 | 309.3022 | Oleoyl Ethyl Amide  (CAS:85075-82-7) | -0.47  (9.3E-3) | 0.11  (5.6E-1) | 0.13  (5.5E-1) |
| Negative | 241 | 413.2593 | Extraneous hits | -0.55  (1.8E-3) | 0.022  (9.1E-1) | 0.12  (5.7E-1) |
|  | 625 | 930.8385 | No hits | -0.53  (2.9E-3) | 0.064  (7.3E-1) | 0.15  (4.9E-1) |
|  | 476 | 640.7291 | No hits | -0.50  (4.8E-3) | 0.031  (8.7E-1) | 0.081  (7.1E-1) |
|  | 451 | 618.7468 | No hits | -0.49  (5.7E-3) | 0.094  (6.1E-1) | 0.14  (5.1E-1) |
|  | 189 | 357.196 | Extraneous hits | -0.48  (7.0E-3) | -0.036  (8.5E-1) | -0.011  (9.6E-1) |
|  | 223 | 389.2561 | N-Oleoyl Taurine (CAS: 52514-04-2) | -0.48  (7.3E-3) | 0.072  (7.0E-1) | -0.024  (9.1E-1) |
|  | 461 | 629.7379 | No hits | -0.46  (9.7E-3) | 0.020  (9.1E-1) | -0.012  (9.5E-1) |
|  | 368 | 559.3425 | No hits | 0.46  (9.9E-3) | 0.051  (7.9E-1) | -0.20  (3.6E-1) |
|  | 435 | 608.3823 | Extraneous hits | 0.48  (7.4E-3) | 0.034  (8.6E-1) | -0.25  (2.4E-1) |
| ^a^METLIN, Metabolite and tandem MS database (<https://metlin.scripps.edu>)  ^b^Data expressed in Pearson r (*p* value) | | | | | | |
